# Identification and validation of specific B-cell epitopes of hantaviruses associated to hemorrhagic fever and renal syndrome

**DOI:** 10.1371/journal.pntd.0007915

**Published:** 2019-12-16

**Authors:** Fernando de Paiva Conte, Bianca Corrêa Tinoco, Thiago Santos Chaves, Renata Carvalho de Oliveira, Janaina Figueira Mansur, Ronaldo Mohana-Borges, Elba Regina Sampaio de Lemos, Patricia Cristina da Costa Neves, Rodrigo Nunes Rodrigues-da-Silva

**Affiliations:** 1 Laboratory of Monoclonal Antibodies Technology, Immunobiological Technology Institute, FIOCRUZ, Rio de Janeiro, Brazil; 2 Laboratory of Hantaviroses and Rickettsioses, Oswaldo Cruz Institute, FIOCRUZ, Rio de Janeiro, Brazil; 3 Laboratório Genômica Estrutural, Instituto de Biofísica Carlos Chagas Filho (IBCCF), Universidade Federal do Rio de Janeiro, UFRJ, Rio de Janeiro, Brazil; Faculty of Science, Ain Shams University (ASU), EGYPT

## Abstract

**Background:**

Orthohantavirus infection is a neglected global health problem affecting approximately 200,000 people/year, spread by rodent hosts and associated to fatal human diseases, such as hemorrhagic fever with renal syndrome (HFRS) and orthohantavirus cardiopulmonary syndrome (HCPS). Circulation of HFRS-associated orthohantaviruses, such as Seoul, Gou, Amur, Dobrava and Hantaan, are supposed to be restricted to Eurasian countries even though their hosts can be a worldwide distribution. Few confirmed HFRS orthohantavirus infections in humans have been reported in American countries, but due to lower medical awareness of the symptoms of this zoonosis, it could be associated to viral underreporting or to misdiagnosis with several tropical hemorrhagic diseases. Serological evidence of orthohantavirus infections, using enzyme-linked immunosorbent assay for the presence of immunoglobulin M and G against recombinant nucleoprotein protein, remains as an essential assay for viral surveillance. In this study, we aimed to identify *in silico* immunogenic B-cell linear epitopes present on orthohantavirus nucleoprotein that are exclusive to HFRS-related species.

**Methodology/Principal findings:**

*In silico* analysis were performed using *Seoul orthohantavirus* nucleoprotein (SHNP) sequence as a model. Linear B-cell-epitopes on SHNP and its immunogenicity were predicted by BepiPred-2.0 and Vaxijen algorithms, respectively. The conservancy of predicted epitopes was compared with the most clinically relevant HFRS or HCPS-associated orthohantavirus, aiming to identify specific sequences from HFRS-orthohantavirus. Peptide validation was carried out by ELISA using Balb/c mice sera immunized with purified recombinant rSHNP. Peptides cross-reactivity against HCPS orthohantavirus were evaluated using immunized sera from mice injected with recombinant Juquitiba orthohantavirus nucleoprotein (rJHNP).

**Conclusion/Significance:**

*In silico* analysis revealed nine potential immunogenic linear B-cell epitopes from SHNP; among them, SHNP_(G72-D110)_ and SHNP_(P251-D264)_ showed a high degree of sequence conservation among HFRS-related orthohantavirus and were experimentally validated against rSHNP-IMS and negatively validated against rJHNP-IMS. Taken together, we identified and validated two potential antigenic B-cell epitopes on SHNP, which were conserved among HFRS-associated orthohantavirus and could be applied to the development of novel immunodiagnostic tools for orthohantavirus surveillance.

## Author summary

Orthohantaviruses are the etiological agents of serious rodent-borne neglected human diseases named as hemorrhagic fever with renal syndrome (HFRS) and orthohantavirus cardiopulmonary syndrome (HCPS). These distinct clinical manifestations of disease are related to specific orthohantavirus species and it is believed that HFRS-associated orthohantavirus mainly circulate into Old World (Asia and Europe) whereas HCPS-associated orthohantaviruses are predominant into New World countries (Americas). However, since *Seoul orthohantavirus*, associated with HFRS, was isolated in America and its natural host (*Rattus norvegicus*) are widely distributed around the world, it raised the question if the viral underreporting is associated to lower medical awareness of the symptoms or if it is associated to misdiagnosis with other tropical hemorrhagic diseases (leptospirosis, yellow fever). In this context, considering that the HFRS are clinically indistinguishable from order hemorrhagic diseases, and that serological tests are predominantly based on serology tests against nucleoprotein, a highly conserved protein among different orthohantavirus, we hypothesize that current available tests do not detect all HFRS-associated orthohantavirus. In this sense; we aimed to identify B-cell linear epitopes exclusively conserved on HFRS-associated orthohantavirus nucleoprotein, using a combination of *in silico* and experimental approaches, to identify targets that could be applied in the development of novel immunodiagnostic tools able to identify different HFRS orthohantavirus species.

## Introduction

Orthohantaviruses belong to genus *Orthohantavirus*, of the family *Hantaviridae*, which comprises a diverse group of infectious negative-sense single-stranded RNA viruses. They are zoonotic in nature and transmitted to humans through the inhalation of contaminated aerosols present in the excreta, saliva, and urine of infected rodent animals [1].

Orthohantavirus infections are a neglected global health problem, affecting approximately 200,000 people/year [1], and are associated with serious and fatal diseases in humans, such as hemorrhagic fever with renal syndrome (HFRS) and orthohantavirus cardiopulmonary syndrome (HCPS). The clinical manifestations of the disease are correlated to orthohantavirus species/genotypes and also it is intimately associated with rodent reservoirs that have a specific geographical distribution worldwide. HFRS is caused by Old World orthohantaviruses such as Hantaan, Amur and Gou virus in Asia and by *Seoul orthohantavirus* (SEOV) worldwide. Besides, orthohantavirus New World species such as *Sin Nombre orthohantavirus* in North America, and Andes, Juquitiba, Laguna Negra, and other related virus in Latin America, such as Juquitiba genotype are responsible for the more severe HCPS [2–5].

Among hantavirus associated to HFRS, the SEOV is spread worldwide by *Rattus norvegicus* and *Rattus rattus*, two rodent species present on the five continents, which are also implicated in leptospirosis outbreaks [6]. In Americas, we highlight that SEOV has already been detected in both rodents, *R*. *norvegicus* and *R*. *rattus*, in Argentina [7], United States [8] and Brazil [7, 9]. Nonetheless, few cases of HFRS have been reported in Brazil [10], where this syndrome could be misdiagnosed as other tropical infectious diseases like dengue, yellow fever and leptospirosis. This data combined with the absence of a specific surveillance for SEOV and others HFRS- associated orthohantavirus in Western countries, and with the inability to confirm cases on site, by laboratory diagnosis, make urgent the development of new diagnostic tools.

In this context, the orthohantavirus nucleoprotein (NP) is the major antigen that elicits early serological responses in infected humans and has been used as a biomarker to develop antibodies for epidemiological surveillance in regions where various orthohantavirus species cocirculate [11, 12]. This protein consists of about 429-amino acid residues, is largely expressed in initial infection and is highly conserved among orthohantavirus [13]. Since the aminoacid sequence of HFRS-associated orthohantavirus are not identical, previous works tried to develop immunochromatographic antibody tests using a combination of different orthohantavirus nucleoproteins [14] or a combination of nucleoprotein and other peptides [15] in order to detect all Eurasian pathogenic orthohantavirus infections. Recently, Kalaiselvan and collaborators suggested that *in silico* predicted B-cell epitopes on NP could be used to identify specific markers to the HFRS [16] and HCPS-associated orthohantavirus [17]. However, the experimental validation of these epitopes remains unexplored. By this way, this study aimed to predict B-cell epitopes on Seoul orthohantavirus nucleoprotein (SHNP), that are exclusively conserved among HFRS-associated orthohantavirus, using a combination of the three most used prediction algorithms, and to validate the epitopes predicted as specific to SEOV or as conserved among HFRS orthohantavirus, against sera of mice immunized with recombinant SHNP.

## Methods

### Sequence data

To predict possible antigenic properties and the three-dimensional (3D) structure of SHNP (Seoul virus BjHD01, Accession Number AY627049.2) using bioinformatic tools, the entire sequence of SHNP (NCBI ID: AAT45728.1) was downloaded from the NCBI website (www.ncbi.nlm.nih.gov/protein) and used for analyses.

### *In silico* prediction of linear B-cell epitopes on seoul hantavirus nucleoprotein

To predict linear B-cell epitopes on the entire sequence of SHNP we used a combination of two prediction algorithms: BepiPred 2.0 (http://www.cbs.dtu.dk/services/BepiPred/) and Vaxijen (http://www.ddg-pharmfac.net/vaxijen/VaxiJen/VaxiJen.html).

Firstly, the prediction of linear B-cell epitopes was carried out using the web server Bepipred 2.0, which uses a Random Forest algorithm trained on epitopes and non-epitope amino acids determined by crystal structures from a protein sequence. For each FASTA input sequence, the server outputs a prediction score for each amino acid. To determine potential B-cell linear epitopes, we utilized the recommended cutoff of 0.5, ensuring a specificity of 57% and sensibility of 59% [18]. Therefore, the Bepipred score represents the average of the scores of at least nine consecutive amino acids above the cut-off. Sequences with BepiPred score above 0.5 were considered as potential linear B-cell epitopes and analyzed by Vaxijen.

VaxiJen is the first server for alignment-independent prediction of protective antigens. It was developed to allow antigens classification solely based on the physicochemical properties of proteins without recourse to sequence alignment. Bacterial, viral and tumour protein datasets were used to derive models for prediction of whole protein antigenicity, showing prediction accuracy from 70% to 89% [19]. To evaluate the antigenicity of predicted epitopes, we used the default cut-off (0.4), suggested to viral antigens. Therefore, sequences with BepiPred score above 0.5 and Vaxijen score above 0.4 were considered potential linear B-cell epitopes and evaluated to specificity.

### Evaluation of conservation degree of linear B-cell epitopes

Sequences identified as potential linear B-cell epitopes were aligned to nucleoprotein sequences of orthohantavirus associated to HFRS or HCPS for similarity comparison. Gou virus (GOUV, Uniprot ID: Q8V6A4); Hantaan virus (HTNV, Uniprot ID: P05133); Amur virus (AMRV, Uniprot ID: A1YQ86), Dobrava-Belgrade virus (DOBV, Uniprot ID: D2IFH1) and Puumala virus (PUUV, Uniprot ID P19475) were the HFRS selected species for the similarity evaluation. Orthohantavirus Juquitiba genotype (JUQV, Uniprot ID A0A248QEV0), Andes virus (ANDV, Uniprot ID: O36307), Laguna Negra virus (LANV, Uniprot ID: C5IAR6) and Sin Nombre virus (SNV; Uniprot ID: Q89462) were the HCPS selected viruses for the conservation degree comparison of the predicted epitopes. All NP sequences were aligned and compared to SHNP and the degree of conservation among them were evaluated by BioEdit Sequence Alignments Editor, Version 7.0.9.0 [20].

### Peptide synthesis

After consensus analysis of the *in silico* prediction tools, the selected sequences were synthesized by fluorenylmethoxycarbonyl (F-moc) solid-phase chemistry [21, 22] (GenOne Biotechnologies, Brazil). Analytical chromatography of the peptide demonstrated a purity of >95% and mass spectrometric analysis also indicated estimated masses corresponding to the molecular masses of the peptides (SHNP_(G72-D110)_: 4211.49 Da, SHNP_(P251-D264)_: 1396.51 Da and SHNP_(I266-A283)_: 2030.32 Da).

### Expression and purification of the recombinant hantavirus nucleoproteins

The cDNA codifying the Seoul or Juquitiba orthohantavirus genotypes nucleoprotein (429 and 428 amino acids, respectively) were cloned into plasmid pET21a(+) to express the nucleoprotein with a six-histidine tag on its C-terminus (Genscript, USA). This construct was used to transform into BL21DE3 strain from *E*. *coli* and the protein was expressed during 4 h upon the addition of 1 mM IPTG. The cells were pelleted by centrifugation at 5,000 *g* for 15 min at 4 ^o^C and stored at -80 ^o^C until use. The cellular pellet was resuspended using the following buffer composition: 50 mM sodium phosphate, pH 6,0, 500 mM NaCl, 1 mM β-mercaptoethanol, 5% glycerol, 10 mM imidazol, EDTA-free protease inhibitor cocktail (Roche, Germany). The cellular lysis was obtained by the addition of 1 mg/mL lysozyme under stirring at 4°C for 30 min, Next, 20 μg/mL DNase and 2 mM MgCl_2_ were added and the solution was incubated for more 30 min. The lysate was sonicated 15 times, alternating 15-seg on-and-off cycles at 4°C. After sonication, the lysate was then centrifugated at 27000 *g* for 20 min to obtain the protein pellet inclusion body. This pellet was dissolved in buffer containing 50 mM sodium phosphate, pH 6,0, 500 mM NaCl, 1 mM β-mercaptoethanol, 5% glycerol, 10 mM imidazol and 8 M urea, overnight stirring at 4°C. The cleared lysate was loaded onto a HisTrap HP column connected to the Äkta Purifier HPLC. Proteins were eluted with a linear gradient of imidazole (10–500 mM) prepared in the same buffer composition and the purified nucleoprotein was refolded by dialysis using a cut-off pore of MWCO of 3,500 Da and the same buffer composition during overnight at 4°C, making three changes using 1-L buffer each time. Recombinant SHNP or JHNP (rSHNP and rJHNP, respectively) were obtained with high purity and yield as analyzed by 15% SDS-PAGE and, as expected, a unique band of about 50 kDa was observed. The refolding process of the rSHNP or rJHNP was confirmed by fluorescence spectroscopy, which was used to monitor the intrinsic tryptophan fluorescence in the buffer containing 8M urea and after the dialysis process.

### Ethics statement

This study was conducted in accordance with the Brazilian National Animal Care Ethical Council. All experiments were approved and permitted by the Ethics Committee for Animal Experimentation of the Oswaldo Cruz Foundation (CEUA-FIOCRUZ, Protocol N° LW-13/16).

### Experimental mice immunization

BALB/c mice (6 weeks old; n = 6) were injected intraperitoneally at day 0 with an emulsion (1:1) containing 50 μg of purified rSHNP or rJHNP, expressed in *Escherichia coli*, in a final volume of 100 μL with complete Freund´s adjuvant (CFA). Fourteen and twenty-eight days after initial immunization, mice were re-injected intraperitoneally with an emulsion (1:1) containing 50 μg of purified rSHNP or rJHNP in a final volume of 100 μL with incomplete Freund´s adjuvant (IFA).

### Enzyme-linked immunosorbent assay (ELISA)

Mice IgG serum titration were assayed at 21 and 35 days after initial immunization using indirect ELISA against rSHNP or rJHNP or B-cell epitopes (SHNP_(G72-D110)_, SHNP_(P251-D264)_ and SHNP_(I266-A283)_). Briefly, MaxiSorp 96-well plates (Nunc, Rochester, NY, USA) were coated with PBS containing 10 μg/mL of rSHNP or rJHNP or synthetized peptides (SHNP_(G72-D110)_, SHNP_(P251-D264)_ and SHNP_(I266-A283)_). After overnight incubation at 4°C, the plates were washed and blocked for 1 h at 37°C with PBS 5% non-fat dry milk (PBS-5%M). Mice plasma samples were three-fold serial diluted in PBS-5%M, starting at 1:50, and added in duplicate wells. After 2 h at 37°C and three washings with PBS-5%M, bound antibodies were detected with peroxidase-conjugated goat anti mouse IgG (Sigma, St. Louis; 1:10000 dilution) and followed by addition of 3,3′,5,5′-tetramethylbenzidine. Optical density was measured at 450 nm using a SpectraMax microplate spectrophotometer (Molecular Devices, Sunnyvale, CA, USA). The IgG serum title was calculated interpolating the value of optical density unit of three times the pre-immune serum (3xPIS) on a linear regression graphic of serum dilution, using the mean optical density of each diluted sera in duplicate.

### Collection of the templates, *in silico* homology modeling and model optimization

The rationale used for template selection and *in silico* homology modeling of SHNP was described by Lima et al. (2016) [23] and carried out with some adaptations. Briefly, for the selection of templates, a search with BLAST and HHBlits [24, 25] using the amino acid sequence of SHNP (GenBank: AAT45728.1) was performed against the expasy SWISS-MODEL template server [26][2][2]. Three structures were selected (PDB ID: 5E04, 5FSG, 4FI5) with the view to obtain as close as possible complete coverage of the amino acid sequence. The server-generated models, which were built and based on the target-template alignment with ProMod3 Version 1.2.0 [27], were used to perform the 2D alignment with the sequence of SHNP and comparative modeling using MODELLERv9.19 by satisfaction of spatial restraints [28]. The lowest energy model was selected using PyMOL Version 1.8 and your 3D structure evaluated with Verify 3D [29, 30] and MolProbity [31]. The energy minimization and correction of Ramachandran outliers was performed manually using COOT Version 0.8.6 [32].

## Results

### *In silico* analysis of Seoul Hantavirus Nucleoprotein (SHNP)

To predict linear B-cell epitopes with potential antigenicity, the full sequence of SHNP (NCBI ID: AAT45728.1) was analyzed by the BepiPred 2.0 and Vaxijen algorithms. As showed in **Table 1**, nine sequences (SHNP_(K30-E48)_; SHNP_(G72-D110)_; SHNP_(G145-K173)_; SHNP_(P182-G196)_; SHNP_(D231-G141)_; SHNP_(P251-D264)_; SHNP_(T347-F361)_; SHNP_(Q363-I380)_; SHNP_(D394-E405)_) presented BepiPred and Vaxijen Scores above threshold values (0.5 and 0.4, respectively) were predicted as antigenic linear B-cell epitopes in SHNP. Among sequences predicted as B-cell epitope by BepiPred 2.0, only SHNP_(E5-V29)_ and SHNP_(D231-G141)_ were predicted as non-antigenic epitopes by Vaxijen and were excluded of further analyzes.

**Table 1:** Potential predicted B-cell epitopes of SHNP.

Predicted Epitope	Sequence	Position	BepiPred Score	Vaxijen Score
SHNP_(E5-V29)_	EEIQREISAHEGQLV	5–29	0.508	0.041
**SHNP**_**(K30-E48)**_	**KQYEKDPDDLNKRALHDRE**	**30–48**	**0.506**	**0.666**
**SHNP**_**(G72-D110)**_	**GKNIGQDRDPTGVEPGDHLKERSALSYGNTLDLNSLDID**	**72–110**	**0.542**	**0.802**
**SHNP**_**(G145-K173)**_	**GRQTSKDNKGMRIRFKDDSSYEDVNGIRK**	**145–173**	**0.511**	**0.652**
**SHNP**_**(P182-G196)**_	**PNAQSSMKAEEITPG**	**182–196**	**0.546**	**1.126**
SHNP_(D231-G141)_	DWTSRIEEWLG	231–241	0.521	0.237
**SHNP**_**(P251-D264)**_	**PIAGSLSGNPVNRD**	**251–264**	**0.519**	**0.617**
**SHNP**_**(I266-A283)**_	**IRQRQGALAGMEPKEFQA**	**264–283**	**0.518**	**1.21**
**SHNP**_**(T347-F361)**_	**TVGTADEKLRKKSSF**	**347–361**	**0.515**	**1.23**
**SHNP**_**(Q363-I380)**_	**QSYLRRTQSMGIQLDQRI**	**363–380**	**0.544**	**0.723**
**SHNP**_**(D394-E405)**_	**DNFHLGDDMDPE**	**394–405**	**0.512**	**1.073**

### The major of predicted B-cell epitopes in HNSP were highly conserved among orthohantaviruses

Nucleoprotein is a conserved protein among orthohantavirus, presenting similarity level ranging from 60% to 99%. However, when compared to orthohantavirus related to HCPS (Juquitiba genotype, Andes virus, Laguna-Negra virus and Sin Nombre virus), the nucleoprotein from orthohantavirus related to HFRS (Gou, Hantaan, Amur, Dobrava-Belgrade and Puumala) were only intermediary conserved, ranging from 62% to 74% of similarity (S1 Table).

Based on this variable degree of conservation of nucleoprotein and aiming to identify immunodominants B-cell epitopes specific to orthohantaviruses related to HFRS, we compare the similarity level of SHNP predicted epitopes among nucleoproteins of HFRS Hantavirus and HCPS Hantavirus. The levels of similarity were classified as very low (<25%;); low (25%>X>50%), intermediary (50%<X<75%) and high (75%<X<100%).

The similarity level observed among the epitopes of each orthohantavirus compared was shown in **Table** 2. Firstly, when compared to other orthohantavirus nucleoproteins, while SHNP presented only intermediary and high similarity levels (62% to 99%), we found B-cell epitopes from very low to high similarity (11% to 100%). The majority of predicted linear B-cell epitopes on SHNP were highly conserved in comparison with orthohantavirus associated with HFRS and to orthohantavirus associated with HCPS. The epitopes SHNP_(G145-K173)_, SHNP_(P182-G196)_, SHNP_(T347-F361)_, SHNP_(Q363-I380)_, SHNP_(D394-E405)_ presented an conservation degree ranged from 67% to 100%, being the sequence SHNP_(Q363-I380)_ the most conserved epitope, with an similarity level of 100% when compared to the most of orthohantavirus associated to HFRS and 94% when compared to virus or isolates associated with HCPS (Table 2). Besides, the predicted epitopes SHNP_(K30-E48)_ and SHNP_(G72-D110)_ presented high conservation degrees (ranged from 84% to 95% and 77% to 97%, respectively) in comparison with to the most of orthohantaviruses associated to HFRS and intermediary degree (ranged from 47% to 53% and 51% to 56%, respectively) when compared to sequences of virus or isolates associated to HCPS. Remarkably, the predicted epitopes SHNP_(P182-G196)_ and SHNP_(P251-D264)_ were considered specific to *Seoul orthohantavirus*, once both presenting 100% of similarity with Gou specie and low level of conservancy (ranged from 14% to 21% and 11%; respectively) in comparison with orthohantavirus associated HCPS.

**Table 2:** Conservation degree of predicted antigenic linear B-cell epitopes among orthohantaviruses.

Related Syndrome	HFRS					HCPS			
Hantavirus / Predicted epitopes	GOUV	HTNV	AMRV	DOBV	PUUV	JUQV	ANDV	LANV	SNV
**SHNP**	99%	83%	83%	82%	62%	64%	65%	64%	62%
**SHNP**_**(K30-E48)**_	95%	84%	84%	95%	47%	53%	53%	53%	47%
**SHNP**_**(G72-D110)**_	97%	82%	79%	77%	59%	51%	56%	56%	56%
**SHNP**_**(G145-K173)**_	97%	90%	90%	93%	76%	86%	86%	83%	86%
**SHNP**_**(P182-G196)**_	100%	100%	100%	93%	80%	87%	87%	80%	80%
**SHNP**_**(P251-D264)**_	100%	36%	29%	29%	21%	14%	14%	21%	14%
**SHNP**_**(I266-A283)**_	100%	61%	61%	72%	17%	11%	11%	11%	11%
**SHNP**_**(T347-F361)**_	100%	87%	87%	80%	87%	73%	73%	73%	67%
**SHNP**_**(Q363-I380)**_	100%	94%	100%	100%	100%	94%	94%	94%	94%
**SHNP**_**(D394-E405)**_	100%	100%	100%	92%	92%	83%	83%	83%	83%

Values express the percentage of similarity of Seoul orthohantavirus nucleoprotein (SHNP) and its predicted epitopes among orthohantaviruses associated to HFRS–Gou (GOUV), Hantaan (HTNV), Amur (AMRV), Dobrava-Belgrade (DOBV) and Puumala (PUUV)—and related to HCPS–Juquitiba genotype (JUQV), Andes (ANDV), Laguna-Negra (LANV) and Sin Nombre virus (SNV). The levels of similarity were classified as very low (<25%; gray cells), low (25%>X<50%; green cells), intermediary (50%<X<75%; yellow cells) and high (75%<X<100%; red cells).

### Animals immunized with HNSP recombinant protein were able to recognize specific B-cell epitopes *in silico* identified in HNSP sequence

**Fig 1:**
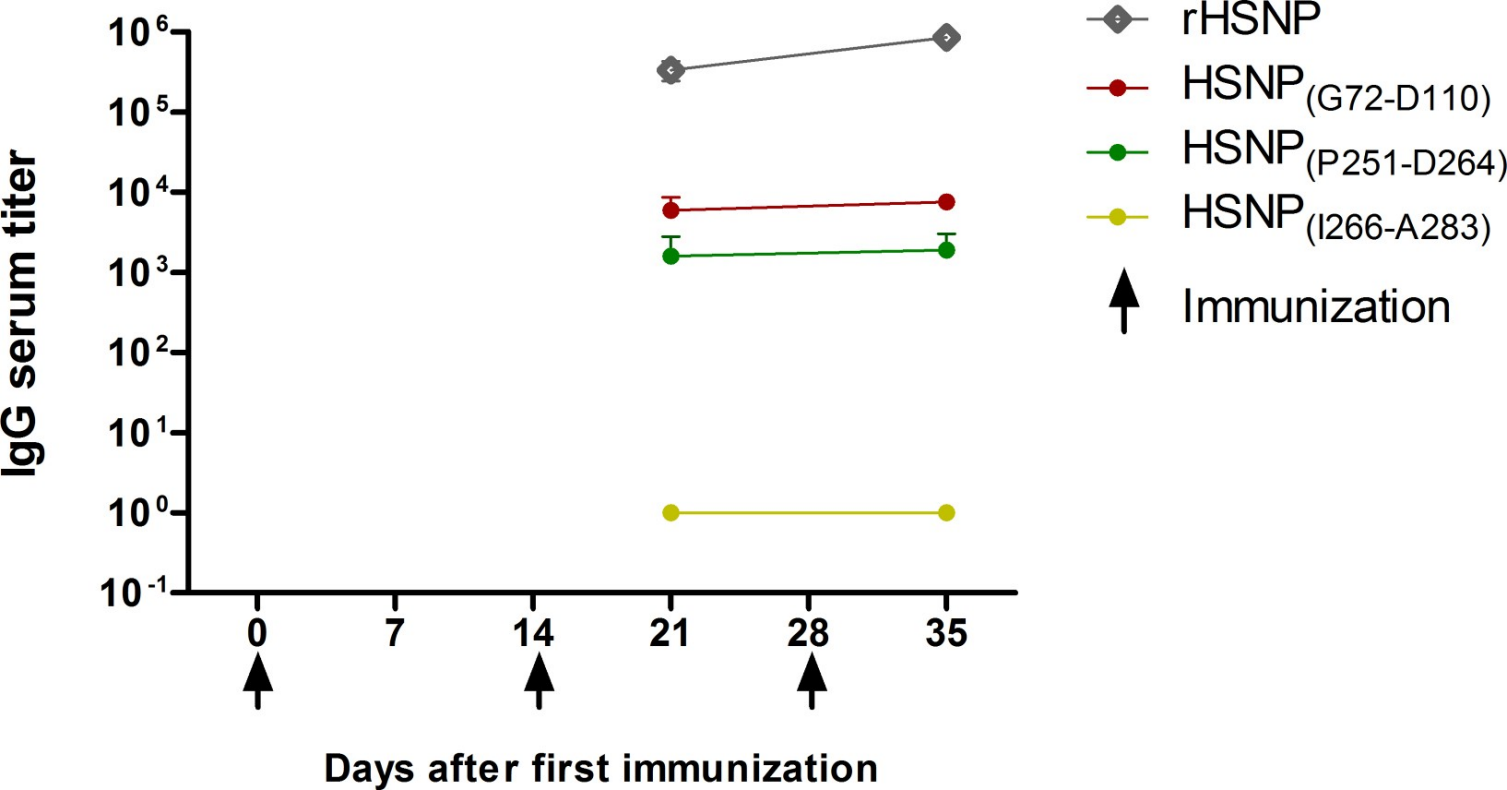
Kinetic of IgG serum title against rSHNP and predicted peptides after experimental immunization with rSHNP. BALB/c mice (n = 3) were immunized with rSHNP emulsified with CFA at day 0 and rSHNP emulsified with IFA at day 14 and 28. IgG serum titer was evaluated by specific ELISA at days 21 and 35 (first and second bleeding). The IgG serum title was calculated interpolating the value of optical density unit of three times the pre-immune serum (3xPIS) on a linear regression graphic of serum dilution, using the mean optical density of each diluted sera in duplicate.

After *in silico* analyzes, sequences of epitopes SHNP_(G72-D110)_, SHNP_(P251-D264)_ and SHNP_(I266-A283)_ were synthetized as linear peptides and tested by ELISA against sera sample from BALB/c mice immunized with rSHNP. This step aimed to validate experimentally epitopes with low (SHNP_(P251-D264)_, SHNP_(I266-A283)_) and intermediary (SHNP_(G72-D110)_) conservation degree among orthohantavirus associated to HCPS. Firstly, we confirmed that rSHNP is immunogenic in animal models because immunized mice presented specific antibodies against it at 28 days after the first immunization (IgG titer: 336829 ± 92407) and presented an increase of 2.5-fold in the IgG serum title against rSHNP after the third immunization (IgG titer: 855671 ± 118257). In relation to the specific humoral response against predicted peptides, mice immunized with rSHNP elicited specific antibodies against peptides SHNP_(G72-D110)_ and SHNP_(P251-D264)_. Both peptides, were specifically recognized, presenting similar levels of antibodies level in the first (anti-SHNP_(G72-D110)_ IgG titer: 5925 ± 2747; anti-SHNP_(P251-D264)_ IgG titer: 1604 ± 1212) and second bleeding (anti-SHNP_(G72-D110)_ IgG titer: 7550 ± 1474 and anti-SHNP_(P251-D264)_ IgG titer: 1911 ± 1119). On the other hands, we did not observe any humoral response against SHNP_(I266-A283)_ peptide (Fig 1).

### B-cell epitopes SHNP_(G72-D110)_ and SHNP_(P251-D264)_ were confirmed as highly specific to *Seoul orthohantavirus*

In order to verify the specificity of predicted B-cell epitopes, the reactivity against rSHNP, rJHNP, SHNP_(G72-D110),_ SHNP_(P251-D264)_ and SHNP_(I266-A283)_ were tested by ELISA against samples from mice immunized with both recombinant proteins, rSHNP and rJHNP and with non-immunized mice, at dilution of 1:100.

**Fig 2:**
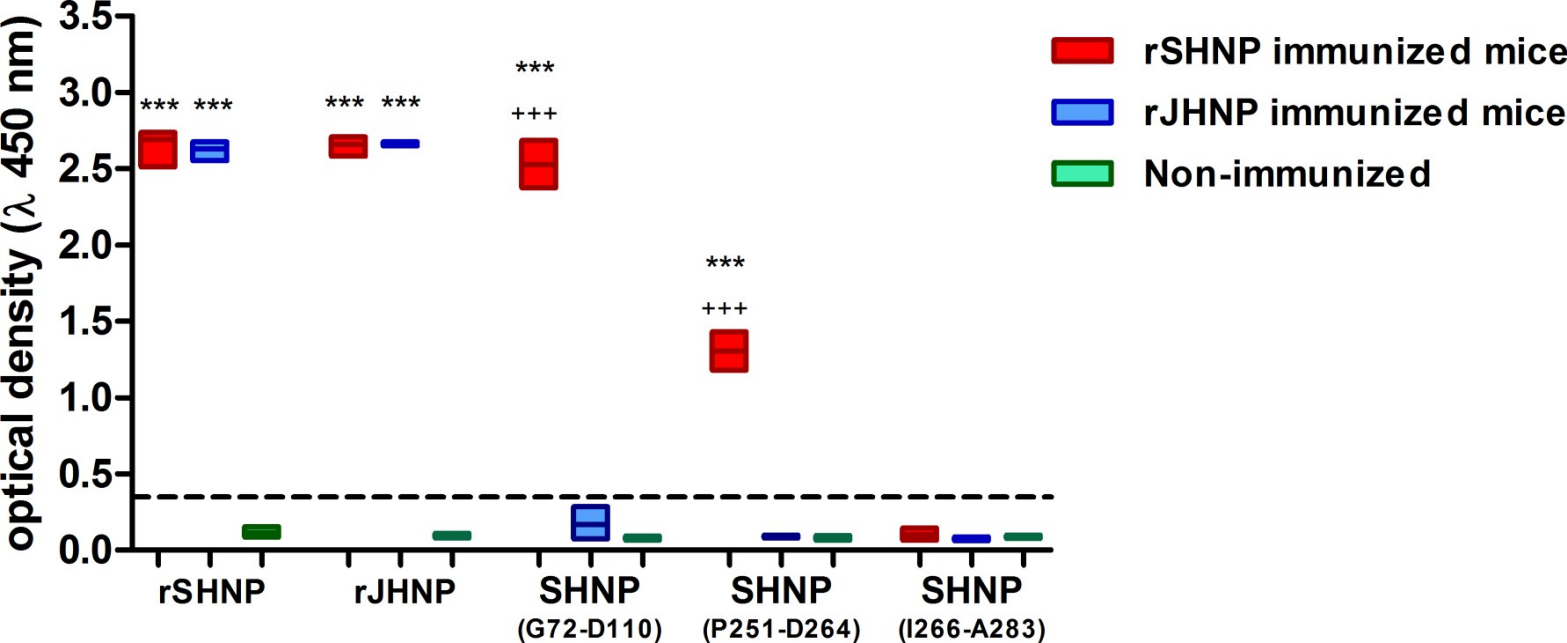
Evaluation of cross reactivity against rSHNP, rJQNP, SHNP(G72-D110), SHNP(P251-D264) and SHNP(I266-A283) elicited by immunization with rSHNP and rJHNP. Bars represent the optical densities of mice immunized with rSHNP (red bars, n = 3) and immunized with rJHNP (blue bars, n = 3) and non-immunized mice (green bars, n = 6). Traced line indicates the value of Cut-off, defined as 3 times the mean of non-immune optical densities. Data is presented as floating bars with lines indicate minimum, mean and maximum values. Values higher than cut-off were considered responder to antigen and compared by test-T against other groups. * represents difference between immunized groups and non-immunized mice, while + represents statistical differences between immunized groups.

Initially, the immunization with rSHNP and rJHNP elicited a high cross reactivity, once that the immunizations results in similar recognize of recombinant proteins, rSHNP and rJHNP, by both immunized groups, immunized with rSHNP and immunized with rJHNP (Fig 2). Despite this, considering the cut off value equal 3times the mean of non-immunized mice values, the epitopes SHNP_(G72-D110)_ and SHNP_(P251-D264)_ were highly specific to SHNP (Optical densities of 2.53 ± 0.17 and 1.3 ± 0.14, respectively) once that non-immunized mice (Optical densities: 0.08±0.011, p<0.0001 and 0.084±0.013, p<0.0001, respectively.) and mice immunized with rJHNP (Optical densities: 0.16±0.09, p<0.0001 and 0.084±0.006, p<0.0001, respectively) (Fig 2). Besides, there no were statistical differences in response to SHNP_(G72-D110)_ and SHNP_(P251-D264)_ between non-immunized mice and rJHNP immunized mice.

### Homology modeling

Search of homologous SHNP structures (GenBank: AAT45728.1) were carried out using the SWISS-MODEL server, using information from Protein Data bank (PDB), and resulted in three protein structures which were used for homology modeling: 5FSG, 4FI5 and 5E04 with sequence identity of 82.91%, 84.62% and 66.08%, respectively. The complete template description is shown in **Table 3**.

**Table 3:** Summary of the templates used for homology modeling of SHNP.

Template	Seq. Identity	Found by	Method	Resolution	Coverage	Description
**5FSG**	82.91	HHblits	X-ray	3.21Å	0.74	Maltose-binding periplasmic protein, orthohantavirus nucleoprotein
**4FI5**	84.62	BLAST	X-ray	2.20Å	0.21	Nucleoprotein
**5E04**	66.08	HHblits	X-ray	2.25Å	0.66	Nucleoprotein

From the alignment of the target sequence with the template sequences, 200 models were built and the lowest energy model was selected. The Molprobity server and WinCoot software was used to evaluate the stereochemical quality of the selected model from Ramachandran plots. After energy minimization, it was possible to reach 97.19% (415/427) of residues in preferred regions, 2.58% (11/427) in allowed regions and only 0.23% (1/427)– 114 GLY—outliers in Ramachandran graph. The 3D structure of the model presented a score of 75.52% on Verify 3D, which demonstrates the quality of the model obtained.

**Fig 3:**
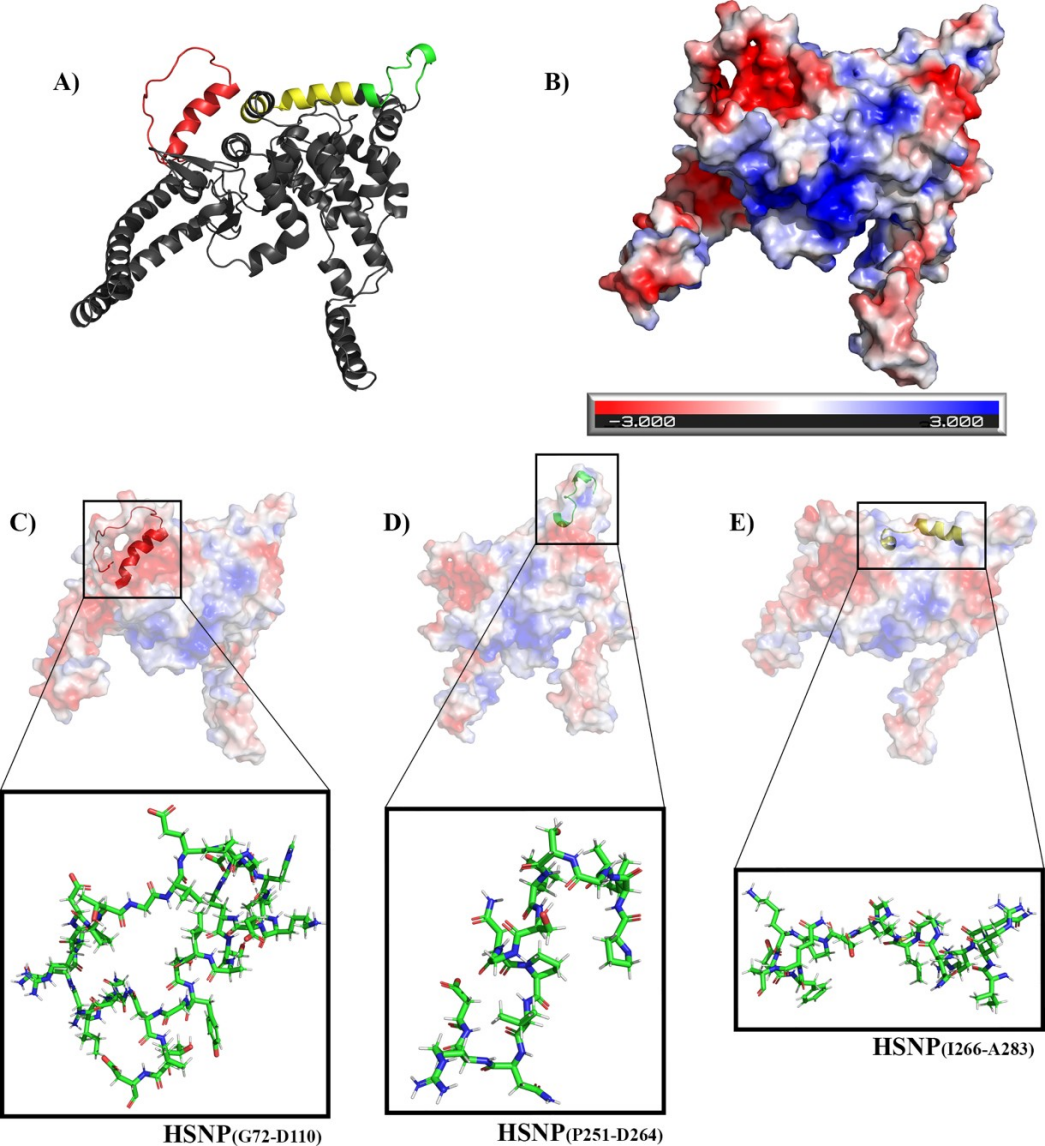
Predicted SHNP homology model. (A) SHNP representation as cartoon with predicted linear peptides spatial localization represented by colors where: red—SHNP_(G72-D110)_; green—SHNP_(P251-D264)_; yellow—SHNP_(I266-A283)_. (B) SHNP electrostatic potential surface representing the positive (blue) and negative (red) charges of predicted protein. Peptides sticks representations of SHNP_(G72-D110)_ (C), SHNP_(P251-D264)_ (D), SHNP_(I266-A283)_ (E) and their respective locations and electrostatic characteristics on the protein surface; SHNP_(G72-D110)_ is in a more electronegative region whereas SHNP_(P251-D264)_ and SHNP_(I266-A283)_ are found in regions with more neutral features.

SHNP homology model and the prediction of its electrostatic surface are shown in Figs 3A and 2B. The spatial location and shape of predicted linear peptides—SHNP_(G72-D110)_ (Fig 3C), SHNP_(P251-D264)_ (Fig 3D) and SHNP_(I266-A283)_ (Fig 3E)—were demonstrated in the model generated. When performing an analysis of the location of the peptides in front of the electrostatic surface of the predicted protein, it is possible to verify that the peptide SHNP_(G72-D110)_ (Fig 3C) is at a more electronegative region (red) than SHNP_(P251-D264)_ (Fig 3D) and SHNP_(I266-A283)_ (Fig 3E) peptides, which are in a more neutral/electropositive region.

## Discussion

Orthohaantavirus species associated with hemorrhagic fevers are worldwide spread; however, they could be misdiagnose as viral or bacterial hemorrhagic diseases, like dengue, yellow fever or leptospirosis, especially in countries where these diseases are endemic. In this context, the development of novel, specific and broader diagnostic tools, able to recognized HFRS-associated orthohantavirus species, have become an urgent demand to public health. Immunodiagnostic tools are vital in the study of orthohantavirus infections due to the limited success with molecular detection as the viremia will be short-lived. Based on this, our study allowed the *in silico* identification and experimental confirmation of two B-cell linear epitopes on SHNP. These data could be helpful to improve the development of new diagnostic tools, resulting in a better and more specific HFRS-associated orthohantavirus diagnose, especially in the New World.

Recently, Kalaiselvan and collaborators suggested that 20-mers peptides *in silico* identified as B-cell linear epitopes may be used for development of geographic region-specific immunoassays [16], however, the experimental validation of predicted epitopes remains pending. Based on this study, we explored the nucleoprotein of *Seoul orthohantavirus*, using a combination of *in silico* approaches to predict antigenic B-cell linear epitopes, non-conserved among hantaviruses associated to HCPS, and to validate its immunogenicity by the reactivity of serum samples from mice immunized with the recombinant protein. Initially, we predicted nine sequences as immunogenic epitopes, in which eight (88.9%) were, entirely or partially, inserted on sequences described in Kalaiselvan’s study [16]. In our point of view, the differences observed between our and his study about predicted epitopes were due the use of BepiPred 1.0, by Kalaiselvan, and BepiPred 2.0, by us. However, the high congruence of both studies reinforces the use of prediction algorithms to identify B-cell epitopes on proteins and remarks the necessity of experimental validation of these results.

Aiming to identify epitopes non-conserved among HCPS orthohantavirus, we compared the conservation of each predicted sequence among orthohantaviruses associated to HFRS and HCPS. The epitopes SHNP_(K30-E48)_, SHNP_(G72-D110)_, and SHNP_(I266-A283)_ were highly conserved just among species associated to HFRS, presenting a conservation level ranging from moderate to low, when compared to HCPS orthohantavirus species. Besides, the epitope SHNP_(P251-D264)_ was considered specific epitope of *Seoul orthohantavirus*, presenting a very high similarity when compared with Gou (100%) and low conservancies (<36%) in comparison with species associated to HCPS or HFRS. Remarkably, all epitopes considered non-conserved among HCPS orthohantavirus (SHNP_(K30-E48)_, SHNP_(G72-D110)_, SHNP_(P251-D264)_ and SHNP_(I266-A283)_) presented low to moderate conservation degrees, ranging from 17% to 59%, when compared with Puumala nucleoprotein sequence. Despite of the association of this orthohantavirus specie with HFRS [33–35], *Puumala orthohantavirus* was also associated with HCPS [36] and is considered the most prevalent pathogen associated with nephropathia epidemica [37], a mild form of HFRS. This epidemics is characterized by acute kidney injury (AKI) and thrombocytopenia [5], suggesting differences among species associated to HFRS. Besides, we observed that major of predicted sequences were highly conserved among orthohantavirus associated to both syndromes, HFRS and HCPS. Our data corroborate Kalaiselvan’s previous studies [16, 17], in which epitopes conserved between both orthohantavirus species, HFRS and HCPS, were *in silico* predicted with prevalence in C-terminal region of the nucleoprotein.

Despite the increasing use of *in silico* approaches to predict epitopes against several diseases, the experimental validation of predicted sequences remains scarce, despite being a critical step to the development of novel vaccines and diagnostic tools. In this context, this was the first study reporting the experimental validation of specific B-cell linear epitopes against orthohantavirus, resulting in the recognition of SHNP_(G72-D110)_ and SHNP_(P251-D264)_ epitopes as promising targets to further development of monoclonal antibodies and/or highly-specific tools to HFRS orthohantavirus diagnosis. Moreover, about the non-reactivity of predicted epitope SHNP_(I266-A283)_ and considering the use of *in silico* strategies to select B-cell epitopes, the oligomerization mode of selected target seems to be an important aspect to epitope selection. Here, we initially predicted three specifics B-cell epitopes in HFRS orthohantavirus and evaluated their localization in tertiary monomeric structure of SHNP, considering all epitopes exposed in protein surface. Controversially, considering that nucleoprotein oligomerizes in dimeric or hexameric forms [3, 38], we believed that the non-reactivity of epitope SHNP_(I266-A283)_ could be explained by its localization in quaternary structure.

Currently, orthohantavirus commercial serological kits are based on nucleoprotein detection however the cross-reactivity among hosts infected by HCPS and/or HFRS-associated orthohantavirus could not be discarded. In our study, we experimentally immunized mice with recombinant nucleoprotein from Juquitiba genotype, a HCPS-associated orthohantavirus that mainly circulates in Brazil [39, 40], eliciting a polyclonal response able to generate antibodies that recognized JHNP and SHNP (**Fig 2**). Based on this cross reaction and considering that Juquitiba genotype nucleoprotein is highly conserved among ANDV and SNV (95% and 86% of similarity, respectively), both orthohantavirus associated to HCPS in Americas, we believe that Juquitiba genotype could be used as HCPS model. Remarkably, despite the cross reaction among JHNP and SHNP, we identify epitopes specific to *Seoul orthohantavirus*. SHNP_(G72-D110)_ and SHNP_(P251-D264)_ epitopes were only recognized by sera from SHNP immunized mice, suggesting that these epitopes are non-conserved among HCPS-associated orthohantavirus and corroborating their potential as HFRS target. This strategy, based on experimental immunization assays using recombinant proteins, have been used to confirm *in silico* findings [41–44], however, considering the probable misdiagnose of *Seoul orthohantavirus* in Americas and the necessity of differential diagnosis of HFRS in New world, experiments using samples of naturally infected hosts (human and/or rodents) should be conducted in order to fully support the present data and to confirm the real potential of these epitopes as differential diagnostic tools.

In summary, we *in silico* identified and experimentally validated the sequences (72)GKNIGQDRDPTGVEPGDHLKERSALSYGNTLDLNSLDID(110) and (251)PIAGSLSGNPVNRD(264) as linear B-cell epitopes on SHNP, which could be helpful to improve the development of novel immunodiagnostic tools against HFRS-associated orthohantavirus species. These epitopes, as synthetic peptides, could be anchored on solid surface or membranes for enzyme immunoassays; or used in the development of monoclonal antibodies, applicable to tools like sandwich ELISA, immunohistochemistry and immunofluorescence tests, which may be relevant to better and more specific HFRS-associated orthohantavirus diagnose, especially in the New World.

## Supporting information

S1 Table Comparison of nucleoproteins similarity among hantavirus. Values express the percentage of similarity of *Seoul orthohantavirus* nucleoprotein (SHNP) and its predicted epitopes among orthohantaviruses associated to HFRS–Gou (GOUV), Hantaan (HTNV), Amur (AMRV), Dobrava-Belgrade (DOBV) and Puumala (PUUV)—and related to HCPS–Juquitiba genotype (JUQV), Andes (ANDV), Laguna-Negra (LANV) and Sin Nombre virus (SNV). The levels of similarity were classified as intermediary (50%<X<75%; orange cells) and high (75%<X<100%; red cells).
(DOCX)
