# Supplementary material for: Identification and validation of specific B-cell epitopes of hantaviruses associated to hemorrhagic fever and renal syndrome
Source: PLoS Negl Trop Dis. 2019 Dec 16;13(12):e0007915. doi: 10.1371/journal.pntd.0007915 (PMC6913923; doi:10.1371/journal.pntd.0007915)
Supplement: S1 Table — Values express the percentage of similarity of Seoul orthohantavirus nucleoprotein (SHNP) and its predicted epitopes among orthohantaviruses associated to HFRS–Gou (GOUV), Hantaan (HTNV), Amur (AMRV), Dobrava-Belgrade (DOBV) and Puumala (PUUV)—and related to HCPS–Juquitiba genotype (JUQV), Andes (ANDV), Laguna-Negra (LANV) and Sin Nombre virus (SNV). The levels of similarity were classified as intermediary (50%<X<75%; orange cells) and high (75%<X<100%; red cells). (DOCX) [file pntd.0007915.s001.docx]

**Supplementary Table 1: Comparison of nucleoproteins similarity among hantavirus**

| **Related Syndrome** | **HFRS** | | | | | | **HCPS** | | | |
| --- | --- | --- | --- | --- | --- | --- | --- | --- | --- | --- |
| **Hantavirus species** | **SEOV** | **GOUV** | **HTNV** | **AMRV** | **DOBV** | **PUUV** | **JUQV** | **ANDV** | **LANV** | **SNV** |
| **Seoul** |  | 99% | 83% | 83% | 82% | 62% | 64% | 65% | 64% | 62% |
| **Gou** | 99% |  | 83% | 82% | 82% | 61% | 63% | 64% | 63% | 62% |
| **Hantaan** | 83% | 83% |  | 96% | 83% | 61% | 64% | 65% | 64% | 63% |
| **Amur** | 83% | 82% | 96% |  | 83% | 60% | 63% | 64% | 63% | 62% |
| **Dobrava** | 82% | 82% | 83% | 83% |  | 60% | 63% | 64% | 63% | 63% |
| **Puumala** | 62% | 61% | 61% | 60% | 60% |  | 73% | 73% | 74% | 71% |
| **Juquitiba** | 64% | 63% | 64% | 63% | 63% | 73% |  | 95% | 90% | 86% |
| **Andes** | 65% | 64% | 65% | 64% | 64% | 73% | 95% |  | 90% | 86% |
| **Laguna-negra** | 64% | 63% | 64% | 63% | 63% | 74% | 90% | 90% |  | 86% |
| **Sin Nombre** | 62% | 62% | 63% | 62% | 63% | 71% | 86% | 86% | 86% |  |

Values express the percentage of similarity among nucleoproteins of Hantavirus associated to HFRS (Gou, Hantaan, Amur, Dobrava-Belgrade and Puumala) and related to HCPS (Juquitiba, Andes, Laguna-negra and Sin Nombre). The levels of similarity were classified as intermediary (50%<X<75%; orange cells) and high (75%<X<100%; red cells).
